# Postcolonial control of Fiji soccer and the return of subjugated knowledges: From the 1970s to the 2010s

**DOI:** 10.3389/fspor.2022.1005733

**Published:** 2022-11-28

**Authors:** Kieran E. James, Henry D. Tuidraki, Sheikh Ali Tanzil

**Affiliations:** ^1^School of Business and Creative Industries, University of the West of Scotland, Paisley, United Kingdom; ^2^Independent Researcher, Nadi, Fiji; ^3^Department of Accounting, University of the South Pacific, Suva, Fiji

**Keywords:** Fiji Islands, Fiji soccer, Foucault, Indigenous Fijians, Fiji Indian, race and class, subjugated knowledges

## Abstract

The primary aim of this article is to use Foucault's idea of subjugated knowledges to search out areas and viewpoints within Fiji soccer which are suppressed by the governing authorities. To fulfill this aim, we explore and assess, *via* ethnographic research, the racial and ethnic aspects of Fiji soccer, from the 1970s to the 2010s, and how cultural hegemony facilitates continued Fiji Indian control and dominance within the sport. Next, and although we note the positive dimension of Fiji Football Association's 2014 Veterans' Dinner, we suggest that some ex-Ba players were apparently discriminated against by, puzzlingly, not being invited. The regulator was also unaware of, or insensitive to, ex-players' transportation needs as some were poor or invalid. We then look at the cases of Sweats Soccer Club (SSC) and Nadi Legends Football Club (NLFC) to show how, in the face of the regulator's indifference to the financial plight of an Indigenous village club (SSC), the ex-Nadi players set up instead a self-help organization (NLFC) to assist and encourage ex-players going through hard times. The latter was a cross-ethnic group/cross-class collaboration between ex-officials and ex-players and was largely outside the regulator's sphere of interest or intent.

## Introduction

Lautoka, Fiji Islands, 2015, 8:00 p.m. on a Tuesday night: My Indigenous friend asks me to give him some money to spend tonight when we go to the pub. I (first author) agree, as it works out better that way. If a white person is spending at a grassroots Indigenous pub, it reeks of British imperialism. If an Indigenous person is spending, the atmosphere in the place is much improved. Renee's is located at the top of a stairway on Lautoka's Naviti Street. Some nights you have to knock on the door to be admitted as there is no-one there but the owner. On other nights, it is packed with revelers, resort workers on their first night ashore, here for a final party before they return to their villages. This night, the owner is seated at a table talking with an Indian woman pub worker. We greet them as we walk past, and the owner heads up to behind the bar, ready to serve. The tables and chairs are wooden and aging and there is a framed map of Fiji on the back wall. It is hot inside the pub, as the place sucks in the daytime heat, so we sit by the open windows and look down at the dark, empty street below. The street reminds me of British road systems elsewhere—the cars only travel one way, while, on the next street over, they travel the other way. It imposes an impression of order on the underlying chaos. The dark shadowy shapes of the Churchill Park grandstands just stand out against the blackness of the night sky one street beyond. The Nadi and Lautoka soccer legend, Henry Dyer, pours out the contents of a Fiji Gold longneck beer bottle into two tiny Fiji-style glasses not much more than two inches high. Draft beer is expensive and not widely available. I feel honored to drink with a soccer hero for Nadi and Lautoka in the national-league and a national team rep in the 1980s. He was in the Fiji team which beat Newcastle United (England) 3-0 in a friendly match in May 1985 and in the team which lost 1-0 to a strong Tahiti side, amidst a player riot, in the 1983 South Pacific Games final. It is both refreshing and sad that ex-players live in practical poverty unlike in the West where they hide behind their mansion walls, thus reinforcing alienation on both sides, as Marx would have said. Such alienation doesn't exist here, only ex-players and ex-managers who will tell you their story over a few beers. Now that the scene has been set, let's begin.

The primary aim of this article is to use Foucault's idea of subjugated knowledges to search out areas and opinions within Fiji soccer which are suppressed by the regulatory bodies. To fulfill this aim, we explore and assess, *via* ethnography, the racial and ethnic aspects of Fiji soccer, from the 1970s to the 2010s, and how cultural hegemony facilitates continued Fiji Indian control and dominance within the sport. Next, and although we note the positive aspects of Fiji Football Association's October 2014 Veterans' Dinner, we suggest that some ex-Ba players were apparently discriminated against by, puzzlingly, not being invited. The regulator was also unaware of, or insensitive to, ex-players' transportation requirements as some were unemployed or invalid. We then look at the cases of Sweats Soccer Club (SSC) and Nadi Legends Football Club (NLFC) to show how, in the face of the regulator's indifference to the financial plight of an Indigenous village club (SSC), the ex-Nadi players set up a new self-help organization (NLFC) to assist and encourage ex-players going through hard times. The latter was a cross-ethnic group/cross-class collaboration between ex-officials and ex-players and was largely outside the regulator's sphere of interest and intent. In fact, the emergence of Fiji Indian cultural hegemony in soccer has led to subjugated knowledge among Indigenous Fijians as well as working-class and less well-connected Fiji Indians (cf. Section Discussion). Their stories and interpretations have been “buried” and hence people have become marginalized from owning their own histories. Lastly, in the Conclusion, we highlight the positive achievements of the Fiji Indian community, both Fiji-based and the Diaspora, in maintaining and running the Fiji soccer senior competitions over the years, despite formidable obstacles.

But, before we discuss soccer, we must introduce two crucial events. In 1874, the Bau chief Seru Epenisa Cakobau (1815–1883) ceded Fiji to the British who remained as the colonial power until 1970 [(1), p. 7]. After gaining power, the British then embarked on a scheme whereby South Asians were brought, no doubt forcibly in some cases, to Fiji to work as indentured laborers on the sugar-cane plantations. Between 1879 and 1916, sixty thousand *girmitiyas* or indentured laborers were brought to Fiji, before the policy was scrapped [(2), p. 14; (3), p. 1; (4), pp. 360, 367; (5), p. 23]. The vast majority of these indentured laborers stayed in Fiji after their terms ended [(1), p. 7]. Their descendants make up the bulk of today's Fiji Indian community (37.5% of the population as at the 2007 Census), although they were joined by Gujarati and Punjabi free settlers during the twentieth century [(2), p. 26; (3), pp. 114–117; ibid., p. 7; (5)]. The first main dynamic in Fiji society is the relationship between the Fiji Indians and the Indigenous Fijians, who were put on a path of separate development by the colonizers [(1), pp. 7, 9–10]. The second main dynamic is the relationship between Western Fiji, centered on the Western Viti Levu (main island) towns Nadi, Lautoka, and Ba, known for the international airport in Nadi and the sugar mills at Lautoka, Ba, and Rakiraki; and the agricultural, pastoral, and timber districts of southeast Viti Levu, which has Suva, the government base, at its center (ibid., p. 4). The British chose to keep the Indigenous people living traditional lifestyles, within village boundaries, while the Fiji Indians moved into the Small and Medium Enterprises (SME) sector, as well as the media, the trade unions, education, and politics, after they gained their freedom. As at the 2007 Census, Indigenous Fijians made up 56.8% of the population, with the remaining 5.7% being Rotumans, part-Europeans, other Pacific Islanders, Chinese, and Europeans.

The fact that the Fiji Indian Football Association became the *de facto* controlling body of the sport in 1938 reflects not only the community's passion for administering, watching, and playing soccer, but also the role they were assigned by the British during colonialism as the merchant class, the intellectual class, the trade unionists, the schoolteachers, and the politicians.

Our overall approach is critical theory-inspired and anthropological. A few quotes from Presterudstuen's (6) book chapter on Fijian men and betting shops in his book on Indigenous Fijian masculinity might prove insightful here. His aim (p. 118) was to look at “how gamblers invest cultural meaning into their various gambling pursuits in particular ethnographic contexts.” He attended betting shops in Nadi town, observed behaviors, discourses, and practices, and studied relationships between individuals in the shop and their responses to gambling, money, winning, and losing. There was a desire not to rely upon preconceptions or other authors' conclusions. For example, other studies found that luck in Papua New Guinea was embedded in a “magico-ritual” traditional understanding of the world. By contrast, the Indigenous Fijians saw betting shops as a part of modernity and separate from Fijian traditional culture.

Knowledge is built up by intensive interaction and continual awareness of context; it is gained through interviews, informal conversations, participant-observation, and written sources. Taxi drivers, students, and colleagues were also important reservoirs of knowledge as many were keen soccer fans. We felt that the immersion of the researchers in the context aided understanding—the first researcher lived in Fiji for 3 years, rather than making short trips in and out of the country. Constant questions were asked, and food and drink were often provided so as to increase willingness to talk and spend longer amounts of time at particular places. Presterudstuen [(6), p. 118] then says that “rather than through the medicalizing or moralizing discourses that dominate academic inquiries into betting, [my approach] enables its theorization as a culturally specific set of practices embedded in social life.” Theorization comes after observation *via* inductive logic. However, our critical theory meant that we aimed to pay attention to instances of inequality and injustice operating within the sport.

## Background

At one stage, and for most of the twentieth century, soccer vied with 15s rugby as the most popular spectator and participant sport in Fiji. During the 1980s, the national soccer team was capable of beating both Australia and New Zealand, on its day, such as the November 1988 match in Nadi when Fiji defeated Frank Arok's highly-favored Australian team 1-0 due to a 67th min goal by Ravuama Madigi (7). This strike has rendered him a legend in Fiji sport until today. The national team ranked around 90 in the world, during the 1980s but, in an alarming fall from grace, the current team has fallen by about 100 places.

How can we explain Fiji soccer's decline, after about 1990, as measured by FIFA rankings and other indicators? The four military coups of 1987 (twice), 2000, and 2006 were very significant and of course, the last one, aiming at multiracialism and meritocracy, was very different from the first three, which favored Indigenous interests in a very partisan way. The coups increased tensions between Indigenous Fijians and Fiji Indians, and homes and businesses of Fiji Indians were targeted by mostly Indigenous youth after both the 1987 and 2000 coups (8, 9). Also, the loss of skilled and talented Indians from the economy, through emigration, has been a negative factor within the country as well as the loss and fragmentation of local memory which emigration causes. The emigration of Fiji Indians has been one reason behind the decline in popularity of soccer and the decline in fortunes of the national team. A second factor was increased professionalism in rugby at the 1987 World Cup and full professionalism, which came in 1995. Successive governments have used the national rugby teams, and especially the 7s team, as a marketing and promotion opportunity and have portrayed it as being a symbol of (Indigenous) Fijian achievement and masculinity—it has been said that it encapsulates the Fijian warrior spirit. A third factor behind soccer's decline after about 1990 was the three-in-a-row title wins of Fiji 7s team at the Hong Kong 7s from 1990 to 19992 with a team which captured the public's imagination [(7), p. 28, (10), p. 18]. This point was made by our interviewee, Henry Dyer. As Dyer explains:

It was the blend of players which gave (the 1990–1992 team) a unique mixture which the other teams did not have [Ratu Kitione Vesikula (coach); Noa Nadruku Tabulutu; Waisale Serevi and the rest of the Nabua boys, including Mesake Rasari, the soldier].

Rugby-league also gained ground by putting teams into Indigenous Fijian villages and overspill areas rather than sticking to the provincial system used by 15s rugby and soccer.

Fiji soccer began as European-controlled, and the game developed along ethnic lines in colonial Fiji under the British colonizers. There were games and tournaments vs. visiting teams and then various *ad-hoc* tournaments and leagues, which were divided along ethnic lines. These contests never included a national competition featuring teams from all over Fiji. In 1938, the Fiji Indian FA (the Fiji Indian-administered body) became the *de facto* national ruling body, and it held the first ever national knock-out tournament, the first Inter-district Championship (IDC) of that year, featuring teams from throughout the island nation. Early predecessor competitions were the club games between a Suva and a Rewa club (Sunshine Sports and Sitare Hind, respectively), which date back to 1922 and were Fiji Indian teams [(11), p. 10]. The Indian Reform League ran an organized club competition in Suva by 1928 (ibid., p. 11), but its weakness was that it had no national reach. In 1961, the word “Indian” was dropped from the regulator's title and in 1962 the national-league opened up to players from all races.

The IDC continued as an annual knock-out tournament, and there were also a number of other cups and trophies contested for, of which only the Jimmy Ram Pratap trophy, contested between Western Fiji powerhouses Lautoka and Nadi, still survives. It is important to note that national-league games were and are only competed for by rival associations and not by clubs. Clubs exist, and are administered by the association body in each particular area. So we have the Fiji Football Association (FFA), the 23 association teams, and then the clubs in the various districts. Some club competitions are inactive or semi-active. Poverty and the distances involved (some populous areas are on the second island, Vanua Levu, and about 100 islands are inhabited) have hindered national expansion and limited clubs to their own districts.

Even by the middle of the twentieth century, Indigenous Fijians were gravitating toward rugby, as spectators, whereas the Fiji Indians were gravitating toward soccer. However, in the 1970s and 1980s, Indigenous Fijians still made up about 75% of national-league players. The provincial market town of Ba in Western Fiji has always been different as its highly successful association team has always commanded passionate support from all ethnic groups and genders in the town and hinterland. It is anecdotally portrayed as the only town in Fiji where soccer is the number one sport and where soccer is discussed 24/7 rather than only on match days.

Until 1977, there was no national-league involving home-and-away league fixtures between district or association teams—there was only the annual IDC and the minor trophies. A national-league, featuring two divisions and promotion-and-relegation, was set up in 1977, involving a limited number of games due to cost and travel times (12). Players mostly worked in other jobs, often working for the sponsor of an association team (such as Ba Motor Parts in Ba). Therefore, the number of games played per year remained fairly small [14 per season from 1977 to 1988, (12)]. In 1978, a new national knock-out tournament for district teams, the Battle of the Giants (BOG), was set up, followed by the Fiji FACT tournament in 1991. Therefore, there are now three knock-out cups, at national-league level, plus the season proper of home-and-away games. There is an extended build-up of media and public interest in the weeks leading up to the knock-out tournaments, and the calendar has now settled down into its contemporary format of the Fiji FACT every May-June, the BOG every July-August, and the IDC (dating back to 1938) every October.

The IDC, in more recent years, is no longer a straight knock-out, but involves group stages of four teams before knock-out begins. The BOG and Fiji FACT have followed suit. The preliminary games are now spread throughout the country, but the semi-finals and final are played in the host-city over the 1 weekend. Like Indigenous rugby-league tournaments in Australia (13), fans head to the host-city for a week or a weekend and enjoy the social atmosphere and the ability to reconnect with old friends, including those who return from overseas as “soccer tourists” [on the “soccer tourist” phenomenon, see James and Nadan (7)]. The national-league first-division is now termed the Fiji Premier League and the second-division is termed the Super League. Traditionally, there was promotion-and-relegation, *via* a two-legged playoff game, between the top team of the Super League and the bottom team of the Premier League. Ba famously avoided relegation at the end of the 1985 season when it defeated Tailevu-Naitasari 7-1 in the first playoff game and both sets of officials and the regulator, in pragmatic Fiji style, decided to call off the second game. The 7-1 game saw Ba stars, Inia “Golden Boot” Bola (Ravuama Madigi's older brother) and Semi Tabaiwalu, both mentally and physically scarred from the motor-vehicle accident which killed Ba captain, Joe Tubuna, in August 1984, make comebacks to steal the game. Now, there is an automatic one-up, one-down system plus a playoff game between the second-top team of the Super League and the second-bottom team of the Premier League. The Premier and Super Leagues have their own concurrent IDCs, and, beginning in 2014, there has been a Masters IDC.

Lautoka Blues dominated national competition up to about 1965, while Ba and Nadi were highly successful in the 1970s and 1980s. Ba, incredibly, won six-in-a-row IDC crowns from 1975 to 1980, under the guiding reins of its legendary manager Sashi Mahendra Singh aka S. M. Singh (1920–1990), while Nadi won three national-league titles from 1980 to 1982 (12). Debate continues today, among taxi-drivers and fans of a certain age, as to which of these two teams was superior. Lautoka, inspired by stars such as Henry Dyer, Sam Lal, Wally Mausio, Kelemedi “Cheetah” Vosuga, and Sam Work, had a short period of dominance from 1984 to 1986 (12, 14), but its progress was halted by the 1987 coup. Nadrogo Stallions, from Sigatoka, had one golden era in the late 1980s and early 1990s, but it has not been successful in more recent times. The capital-city sides, Rewa and Suva, and Labasa from Vanua Levu also sometimes challenge for title honors. Rewa played in the 2022 Oceania Champions League in New Zealand (won by Auckland City).

## Materials and methods

This research project commenced in early 2014 when I (first author) was living in Western Fiji and met Mr. Bobby Tikaram, former president of both Airport Soccer Club and the Nadi Soccer Association, and the star central-midfielder for Nadi and Lautoka and the Fiji national team, Henry Dyer, whose career spanned the 1980s and early 1990s. To retrace my footsteps slightly, I arrived in the Fiji Islands in May 2013 to take up an academic appointment in the Western Fiji region and, during the course of that year, I became a supporter of Lautoka Blues in the Fiji Premier League, and an attendee at most home games at Churchill Park Stadium. I was interested to pursue research into Fiji soccer and so my meetings with Tikaram and Dyer were fortuitous. After initial discussions, it was agreed that I would co-create Dyer's memoirs and was given freedom to publish academic articles from the primary data.

The overall approach here is critical theory-inspired and anthropological, with most primary data having been gathered through oral history-style interviews and informal conversations. There was also participant-observation, which I will explain shortly. I met Dyer 20 times, for an average length of 3 h per meeting, between May 2014 and April 2015, for the purpose of writing the memoir book. Dyer began at the first meeting recounting his background, tracing it back to the arrival of the Dyers, sandalwood traders from Yorkshire, several generations ago, before moving on to his childhood and teenage years, and ending with his senior team debut in 1981 for Airport Soccer Club and then the Nadi Soccer Association team. At other meetings, we began by looking at some key matches Dyer had played in, while partly following a chronological basis, so that his post-retirement years came last. He would either introduce a new topic at the meeting itself or we would agree on the topic at the previous meeting. I asked clarification questions, and these sessions could best be called unstructured interviews because, as they were one-off, there was no standard set of questions.

The second stage of the research, conducted over the period June to October 2015, involved interviewing five ex-Ba players and two ex-Nadi players (in addition to Dyer) as well as Bobby Tikaram and Nadi Association team doctor in the 1980s, Dr. Raymond Fong. All but two of the ex-player interviews were 3 h long. Dyer participated in all but one interview. Interviews took place at the players' homes, at the Ba River foreshore, the Ba Central Club, or the Ba rugby ground. One ex-Ba player, Julie Sami (not a person born as a woman or a trans person despite the name), and one-ex-Nadi player, the late Vivekanand “Boy” Reddy, were Fiji Indians while the others were Indigenous Fijians. “Julie Sami” is an alias he used in his soccer career. The ex-players' wives participated spontaneously at three of the interviews and their words were recorded. These interviews were semi-structured as a standard set of questions was raised deliberately each time [on semi-structured interviews, see (15)].

Interviewees were chosen largely through a combination of purposeful sampling, convenience sampling, and snowball sampling. Dyer and I decided to focus on Ba and Nadi ex-players from the 1980s as Dyer lived in Nadi and had strong connections with ex-Ba players. Furthermore, Ba and Nadi were the top two teams in the first half of the 1980s and Ba is within easy traveling distance from both Nadi and Lautoka on a weekday. We had wanted to interview at least one Fiji Indian player, to get a mix of perspectives and to avoid accusations of bias, and we achieved this goal. We avoided players now resident in Suva or Vanua Levu, as such trips require overnight stays and can be expensive. My time away from my then university was limited as leave was not permitted during teaching weeks. It is a limitation of the study that no Suva, Rewa, Nadroga, or Labasa ex-player was interviewed. However, we interviewed 6 out of 22 (27%) of the starting XIs from the Ba-vs.-Nadi 1982 IDC final, which is a fair achievement, given that some ex-players have died or emigrated.

Of the interviewees, our first interviewee, Meli Vuilabasa, is an ex-Ba and Fiji defender and presently an influential person in Indigenous Christian circles both within and beyond his home village (Natalecake, pronounced Natale_tha_ke_as). He comes across as more serious and restrained than the others, due perhaps to his strong Christian beliefs. As this was our first interview with an ex-player other than Dyer, we were possibly also slower and more structured in our approach than in later interviews where a rapport had already been established due to the word being passed back by the earlier interviewees. The second interviewee was ex-striker for Ba and Fiji, Inia Bola, who was mentally scarred by the motor-vehicle accident of 1984. He stumbled on his words and his thoughts occasionally and we and his wife helped him out where we could without putting words into his mouth. He apparently believes that his children died because of black magic by opposing fans, and it is hard to know how to approach such a belief. Third was Semi Tabaiwalu, humble and thoughtful, but more rebellious due to his experience of not being rehired by Ba as manager despite trophy successes. He was very outspoken and direct, using analogy, sarcasm, and forthright comments. We began at Ba River foreshore, a place of supporters' parties after Ba title wins in the 1970s, then moved on to Ba Central Club.

Fourth interviewee, Savenaca Waqa (pronounced Sava_natha_Wang_ah), was a famed and esteemed goalkeeper for Nadi in the 1980s and first-choice keeper for Fiji when the German Rudi Gutendorf (1926–2019) was manager. He was there with his protégé, later Nadi goalkeeper, Seremaia Tale. Both were restrained and not overly wordy or demonstrative, but Savenaca exerted a strong and calm presence. One of his greatest ever efforts was the clean sheet he kept in the 1985 friendly match against Newcastle. At our interview, he was reflective and circumspect when asked about that game: “Now, when I see them on the TV, I remember that time when they came to Fiji. It takes my memories back. I am amazed today that we could play at that level. That is the history.” He is sad nowadays at the falling standards of Fiji soccer. The fifth interviewee, in October 2015, was the working-class Fiji Indian, Julie Sami, who was a wiry midfielder for Ba and Fiji, and older brother of Vimal. We interviewed him on the porch of his house in the Ba countryside and his wife exchanged banter with us through a window. He was tough and cynical, but very humble and grounded. He too is dismayed at Fiji soccer's decline and the way his son was treated as a player for Ba. Dyer distinguishes the working-class Sami brothers, who mixed with Indigenous boys in Ba and later at Fiji Sugar Corporation (FSC), from Fiji Indian players, such as Farouk Janeman and Vimlesh Singh, who belonged to business-house families and maintained separation. This was not a personal criticism of Janeman and Singh, more a piece of sociological observation, with the benefit of 30 years hindsight.

Throughout the research, I maintained awareness of the strengths and weaknesses of the oral history approach. If we consider limitations of oral history first, Huang et al. (16) look at some oral history research into traditional Chinese martial arts in China and conclude that problems include: lack of honest disclosure by participants; participants wanting to delete content after interviews because of fear; insufficient number of quotations cited; lack of verification with outside sources; biased recounting by interviewees so as to overstate the importance of masters from one's own school; and authors' over-reliance on interviewees with a lack of authorial interpretation and authoritative voice. The strengths of oral history are that we can preserve a record of how ordinary people lived, interacted, and perceived their lives, which continues on after their passing, and our record can give voice to marginalized voices and underclass persons who are frequently excluded from the official record.

In terms of participant-observation, I attended numerous village functions and drinking sessions in town. I attended the 2014 FFA Veterans' Dinner held in October 2014. I was first asked to leave this meeting by a young Fiji Indian man. On telling Dyer that, for the sake of the research project, I hoped to stay, he approached the FFA president, Mr. Rajesh Patel, who was at the lectern and about to speak. Dyer says that the conversation proceeded as follows:

Henry Dyer: My friend, the researcher, wants to stay for the dinner, but he was asked to leave.
Rajesh Patel: I didn't organize that.
Henry Dyer: But your name was mentioned.
Rajesh Patel (anxious to start the meeting): OK, he can stay.

Participant-observation also included my observing, photographing, and then later writing down what happened, plus my interpretation, at the beer-drinking episode with Dyer and Tabaiwalu at the Ba Central Club. I always adopted the inquisitive and calm attitude of wanting to watch and learn, and I always took my pen, notebook, and camera along with me.

## Theory

### Subjugated knowledges in Fiji soccer

Foucault [(17), p. 81] explains two aspects of subjugated knowledges: Firstly, he means the “historical contents that have been buried and disguised in a functional coherence or formal systematisation.” The phrase “historical contents” is important as it des not distinguish between facts and theory (hence Foucault himself was accused of neo-functionalism). Although Fiji Premier League players may now get paid cash, unlike in the 1980s, they are still underpaid and given no major importance or power in the grand scheme of things. In a famous counter-hegemonic statement, a Fiji Indian lawyer, Iqbal Khan, of Lautoka, stated that the FFA treats players like tea-bags, a cute analogy in a former British colony, i.e., they are used up during their playing careers and then forgotten and cast aside (source: Henry Dyer, online communication to author, May 28^th^, 2022). In the “functional coherence or formal systematization” (p. 81), these subjugated statements are forgotten or don't get a hearing. They serve no *function* in terms of running regular competitions, promoting the sport, or securing sponsors for the sport. Therefore, they are excluded from FFA discourses such as media statements and glossy tournament publications. But sometimes ex-players are spotted in or outside the grounds, dressed shambolically, and with little money for beer, food, or even admittance to the ground. Even their very presence can be viewed as uncontrollable subjugated knowledges. Often younger fans do not know who they are, and the media does not always care. Their aging bodies mirror the decrepit wooden grandstands, which are always-already halfway falling apart at many of the Fiji Premier League grounds. As Foucault [(17), p. 81] says, about his writings on the asylums and prisons, what they really amount to is “the immediate emergence of historical contents” and these contents are designed to stimulate and provoke rather than pacify or reassure. In Foucault's [(17), p. 82] words, “[s]ubjugated knowledges are thus those blocs of historical knowledge which were present but disguised within the body of functionalist and systematizing theory and which criticism … has been able to reveal.”

The second aspect of the idea of subjugated knowledges are those deemed inadequate to their task or deficient or “naïve” (p. 82) in some respect—they are perceived as “located low down on the hierarchy” (p. 82) of knowledge, of failing to make the grade. These directly disqualified knowledges often come from low-status or marginalized individuals who have little or no authority to make official statements or history on behalf of this or that body. This would include, says Foucault, psychiatric patients, ill persons, nurses, doctors, and delinquents. “The regulation of discourse,” writes Stoddart [(18), p. 205], “deals with who is allowed to speak on a given topic.” While the high-status lawyer, Iqbal Khan, had knowledge that was buried, other types of knowledge are even more marginalized due to the status of their speaker. Our interviewed working-class and underclass (mostly Indigenous) ex-players also produce knowledge not valued by the regulator, the commercial world, or the media. They are different from the spokesmen used by those sources who are usually well-presented, employed in full-time jobs, and from business-house families. Even the humble General Practitioner, Dr. Raymond Fong, could be viewed as part of this category since his unassuming manner, Chinese ancestry, and the fact that he practices alone in Nadi distance him from current sources of power in soccer (as opposed to medical) circles. The fact that he was Nadi team doctor in the 1980s is largely forgotten knowledge, remembered only by the people who were intimately involved. His view (source: personal interview with author, July 24^th^, 2014, Nadi town, Ba province, notes in possession of author) that Henry Dyer was a better player than Fiji's most famous soccer export, Roy Krishna, who now plays in the Indian Super League, will fall through the cracks of systematized and approved knowledge because it contradicts modern marketing logic.

**Figure 1:**
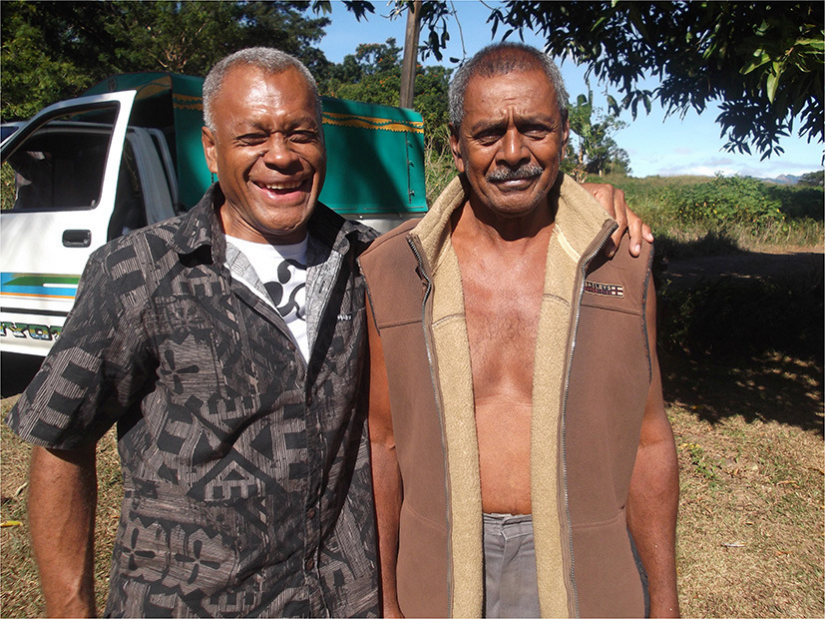
Henry Dyer **(left)** with Semi Tabaiwalu, Ba, Fiji, 17 June 2015.

Other literally local and subjugated knowledge brought to light by our research was the harshness of Ba Soccer Association powerbrokers in not rehiring Indigenous Fijian manager, Semi Tabaiwalu, after a successful 4-year stint in which he won each of the four available trophies at least once. We brought this point to light on our website and in other published articles. To cite Foucault again, “it is through the re-appearance of this knowledge, of these local popular knowledges, … that criticism performs its work” (p. 82). Figure 1 shows ex-Fiji stars Henry Dyer (left) and Semi Tabaiwalu in Ba on Wednesday, June 17^th^, 2015.

## Discussion

### Introductory remarks

The main part of this section begins with the heading “Ethnic and Racial Aspects.” Then we have subsections on the Veterans' Dinner and the Nadi Legends Football Club. The dinner example is a specific example of the FFA's lack of understanding of the practical difficulties that poor and invalid players would face in attending the dinner. There is also the potential discrimination issue in terms of some players being invited and some not. Then the Nadi Legends Football Club (NLFC) subsection shows ex-officials and ex-players responding to difficulties by forming a self-help organization. So this subsection is placed third here. It contrasts the NLFC with the earlier Indigenous Sweats Soccer Club (SSC), which failed for financial reasons and received no support, moral or financial, from the regulators. We point out how. by not fielding an actual team, NLFC positions itself outside of the regulator's area of intent. The ex-player-created Jone Nakosia Veterans' Tournament of 2012–2013 then saw the regulator (the FFA) jump into action by launching its own Masters IDC in 2014.

### Ethnic and racial aspects

The Italian Marxist Antonio Gramsci [(19), p. 12] defined hegemony as “the ‘spontaneous' consent given by the great masses of the population to the general direction imposed on social life by the dominant fundamental group.” Jackson Lears [(20), pp. 569–570] adds that: “The available vocabulary helps mark the boundaries of permissible discourse, discourages the clarification of social alternatives, and makes it difficult for the dispossessed to locate the source of their unease, let alone remedy it.” In a discussion of Gramsci's concept of hegemony, Jackson Lears (20) reinforces that it involves a group, united perhaps by not only economic interests, but cultural or religious ones, being able to form a historic bloc which then vies for hegemony. It may attain hegemony if it can persuade others that its views are universal views and that it speaks for the whole of the society [(18), p. 201]. Contradictory consciousness may exist where the working-class simultaneously exalts some aspects of dominant values while also ridiculing them as not accurately reflecting the realities and struggles of their lives [(18), p. 209]. For Jackson Lears (20), language is a pivotal part of hegemony where subordinated groups lack the language and opportunities to speak forcefully in a way which resists and opposes the dominant group. The dominant group is able to deprive them of language and space which would render their ideas capable of being thought and said in a way which would not lead to immediate rebuttal.

Fiji Indian cultural hegemony is achieved through the expression of dominant discourses and practices, through institutions of various types, and through religious observance, in such a way that existing social hierarchies are maintained and reproduced. This impacts upon lower-status Fiji Indians and some Indigenous Fijians either directly or through reduced life-chances and opportunities (where the cause is not obvious). Fiji Indian hegemony implicitly rests on the legitimacy and authority of the moral suffering of the *girmitiyas* as well as the moral *gravitas* of the anti-colonial struggles in both Fiji and India. Blame for the coups is also placed on the Indigenous people and hence the blame for emigration. Another factor contributing to the ability of the Fiji Indians to gain cultural hegemony in a particular sphere of life (such as the SME sector or soccer) is the successful containment of Muslim-Hindu antagonisms due to the *girmitiya* narrative. Also important is the constant reverential remembering of Fiji among the Diaspora, which allows Fiji Indians to maintain a separate identity from other Indians in the West (and in India) and helps maintain hegemony within Fiji. “Would the last person to leave please switch off the lights,” but it must be them that does the switching. The Fiji Indians also try to project an impression that their management style is the most effective and efficient. To some extent, this view has been internalized by subordinated groups such as the Indigenous.

Sometimes Fiji Indians seem to provoke or challenge the Indigenous Fijians to choose between loyalty to the British or to the contemporary power structure (Fiji Indian power or perhaps Bainimarama's Fiji First party). This is a deceitful and cunning maneuver as it does not represent a real choice in contemporary Fiji—the British are never coming back (as a colonial power) so it is deceitful to imply that this is a real option when it is not.

**Figure 2:**
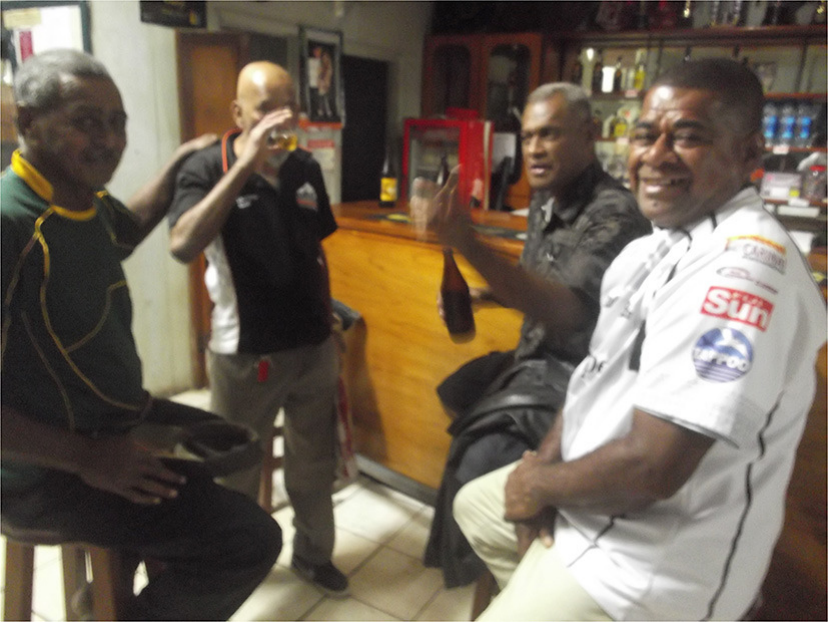
Barman accepts drink from Henry, Ba, 20 June 2015.

At a traditional Indigenous Fijian kava ceremony, if a person receives the full bowl from the server, she/he is expected to drink it in one go and then pass it directly back to the server without it touching the floor or a table (6, 21). This is a symbol of reconciliation, forgiveness, and fellowship. Sometimes it is replicated at a beer-drinking session, and the meaning stays intact if that is the people's intentions [(6), p. 103]. Presterudstuen [(6), p. 103] argues that beer-drinking sessions “assuage social hierarchies,” rather than remove them, and he points out correctly that a more informal atmosphere operates at beer-drinking, as opposed to kava, sessions. Henry Dyer and the first author took Semi Tabaiwalu into the Ba Central Club on the late afternoon of Saturday, June 20th, 2015. Because this club has a connection with Ba Soccer Association, and is a favorite drinking spot of FFA president, Rajesh Patel, Tabaiwalu was nervous and circumspect as his memory was haunted by the time when he was pushed out of his managerial (i.e., head coach) role at Ba Soccer. We hoped that our enthusiastic presence would allow him to hold his head high again in this place and have his self-confidence restored. Dyer poured a glass of beer from a Fiji Gold longneck bottle, and handed it to the Fiji Indian barman who, understanding the protocol, responded by downing the contents of the small glass and handing it back to Dyer (see Figure 2). We interpreted this act of drinking as Tabaiwalu publicly being granted back honorary status within Ba Soccer in the presence of witnesses. Presterudstuen [(6), p. 101] uses the term “stylized insolence” to describe social practices “used by many men to challenge traditional notions of masculinity.” Arguably, we observe “stylized insolence” mixed with mutual respect here as the insolence takes on a humorous turn in order to blunt its force—there is an aspect where the practice described is a clear protest against Fiji Indian cultural hegemony within soccer. Without the laughter and jesting (see Figure 2), the “protest” would probably have been deemed unacceptable or even offensive. Tabaiwalu perceives that he was pushed out of his job by the Ba Soccer Association powerbrokers, despite his considerable success in winning trophies, because he was too outspoken. This may have challenged the ability of the Fiji Indian leadership to keep control and order by the traditional means of cultural hegemony and benevolent, paternal acts of kindness mixed with control. The following conversation occurred at our interview with Tabaiwalu on June 20^th^, 2015:

Semi Tabaiwalu: From 2007 to 2010 (4 years) I was coach of Ba team and I scooped all the titles [Authors' note: Ba won the Fiji FACT in 2007 and 2010, BOG each year from 2007 to 2009, IDC in 2007, and the national-league title in 2008 and 2010.]
Henry Dyer: So that would have been one of your best soccer achievements in management.
Henry: Why did you pull out of Ba Soccer as a coach?
Semi: Because of the management.
Henry: Did they drop you as a coach?
Semi: Yes, they said my term is over. I knew already because every time I go against them I always talk straight to them about how things should go.

If we call the fact (i.e., the dismissal) and the criticism of the fact together as an outburst of subjugated knowledge, then the practice of beer-drinking at the Central Club enacted that knowledge as spectacle in public view. This was able to happen as first Dyer and I raised up the status of Tabaiwalu and then the meaning inherent in a kava ceremony was seized upon and brought to bear in such a way that all present understood that the meaning had stayed intact, despite the fact that the beverage was beer, not kava, and it was outside a village setting. Thus, we conclude that the episode which ended with the barman's beer-drinking can be understood as a ceremony rather than as purely a simple social engagement.

A further way to understand this event is *via* the concept of liminality [(22), p. 96] where a liminal being is “neither one thing or another; or maybe both” all at the same time. Parreñas [(23), pp. 84–88] gives the example of transgender Filipina hostesses at transgender pubs in Japan, where they are both not-men and not-women at the same time (as many do not want the necessary surgery and they recognize that their life experiences are different from those born as women). However, they can also be seen as “both” due to their exaggerated displays of femininity, during their nighttime dances, and the awareness of customers that a transgender pub is intrinsically different. To put it another way, they are both women and not-women. In fact, these knowledges are part of the allure and attraction for customers. Similarly, the beer-drinking episode both is and is not a kava-drinking ceremony. The ritual is veiled, humorous, cheeky, mystified, but still real once the event has taken place in all its glory. It would verge on the undeniable in the way it was conducted on that day, but the humor and the pub context saved it from being confrontational as in “why on earth did you fire the poor guy?”

A Foucauldian analysis here would also rely on his notion of surveillance and control leading to the regulatory gaze and self-discipline [(24), p. 167]. In a way, the gazing can be reversed [(24), p. 167], as in when the ex-players judge the action and inaction of the Fiji Football Association and Ba Soccer Association. “Therefore, networks of power/knowledge,” writes Stoddart [(18), p. 206], “are also sites of resistance, where all of the partners within a power relationship produce and contest the truth.”

Furthermore, Tabaiwalu cites the case of the late George Koi, the only or at least the most conspicuous appointment of an Indigenous Fijian to an official post at the FFA. He was soon pushed out of his post, again, as in Tabaiwalu's case, by being allegedly too outspoken and too willing to investigate matters which other officials preferred to lie buried. “We don't know all these things about the economic and monetary side of playing dirty,” said Tabaiwalu to the author. Tabaiwalu added: “This always enabled the Indians to run the show and we let them run the show.” However, one positive thing we should note is that the Fiji Indian emigrant community often pays for Tabaiwalu to visit New Zealand and coach the Ba team in the replica IDC tournament which is contested in New Zealand among the émigrés.

The discussion here at the same interview revolves around George Koi:

Author: What is your comment about the fact that Indigenous Fijian players seem to find it harder to become coaches and officials than Fiji Indian players do?
Semi: Because us [Indigenous] Fijian boys we are very good at heart. We work straight, we talk straight; we don't know all these things about the economic and monetary side of playing dirty. This always enabled the Indians to run the show and we let them run the show. A good example is about our former friend and player George Koi. He was the first Fijian (apart from the Indians) to become a Vice-President of Fiji Football. This may have been in the late 1990s to the 2000s, I cannot remember exactly. In his first year, he started to find about how Fiji Football was working. He found out certain detailed facts about the Association and they were worried about this guy. He was removed after 3 months in office.

Later, at the same interview, after we had moved from Ba River foreshore to Ba Central Club, Dyer spoke as follows about Tabaiwalu's exit from Ba Soccer Association:

Henry: The Indians, they have the power, they manipulate, they have the monopoly. Regardless of your experience or ability, when they say “time,” then your time ends there. Because they control the game.

This is probably the clearest statement yet about the reality of Fiji Indian hegemony within soccer and how it works in practical terms. We see here use of the colloquial, everyday, marketplace terms, “Fijians” and “Indians,” rather than the politically correct terms “Indigenous Fijians” (or “iTaukei”) and “Indo-Fijians.”

Another ex-Ba and Fiji player, Meli Vuilabasa, was asked by the author why few Indigenous Fijian spectators are seen at Premier League games (outside of Ba town) whereas, in the 1980s, all ethnic groups were strong and active supporters at district games. Calm, thoughtful, and reflective, the deeply Christian Vuilabasa argues that the Indigenous people are better educated now and that even those who are not can see the metaphorical lie of the land and realize that “the sport does not belong to them.” Often the only Indigenous spectators seen at games now are relatives or friends of the players. He points to the lack of Indigenous Fijians serving as officials at the level of the FFA or the district level, and the lack of Indigenous Fijian managers. Although 75% of players in the 1980s were Indigenous Fijians, while the remainder were Fiji Indians, the ratio is reversed for managers with six out of the eight Premier League managers being Fiji Indians in both 2015 and 2019. No advances were made in this area between 2015 and 2019 as, although the faces changed in the interim, the ratio stayed the same.

At the interview with Vuilabasa at his house in his village just outside of Ba town on June 2nd, 2015, Vuilabasa answered the author's question as follows:

Author: Nowadays we see very few Indigenous Fijians at district games but Henry [Dyer] tells me it was different in the 1980s. Why is this and can it be changed?
Meli: It can be changed, [author's first name]. There are so many Fijians who are educated now. When they see things they can make out what is happening. Even the uneducated can understand. In Fiji Football there is not a single administrator who is [Indigenous] Fijian. At the district level there is only a few. Of the eight districts there are two Fijian coaches and few or none in the administration. The people feel that the game does not belong to them.
Henry: They feel left out.

A further short discussion occurred later in the interview as follows:

Author: And you don't see the Fijian boys these days playing soccer in the village.
Meli: Probably for the same reasons you have given. They feel excluded from the game. Many Fijians especially are interested in playing rugby now. They are better looked after and get recognition.

Julie Sami also expresses a concern that “there is a racial feeling in the game now” and that “it is not good for the sport,” which presumably refers to the control of the sport by the Fiji Indians and the cultural hegemony needed to secure continued domination and adherence. He also says that the FFA only communicates information about training courses for coaches through insider networks rather than advertising them through the mainstream media or FFA publications. This amounts to class-based discrimination and hegemony, given Sami's working-class roots as an Indian who had many friends among the Indigenous youngsters growing up who later worked alongside him at the FSC in Ba. He was an FSC employee at the time of Tubuna's death in 1984 and he had a premonition dream about the death. At the 2015 IDC finals in Ba, Sami showed me a copy of a small, business-card sized card which granted him free admission to each day's session at the IDC, but he lamented that it did not cover admission to other home games or to away IDCs. This was an example of how Sami perceived that the FFA was not showing due care toward the ex-players given their circumstances.

All the material presented here can be viewed as subjugated knowledges because the facts and interpretations were buried or absent from approved oral discourses and written publications. They present an alternative view of how Fiji Indian cultural hegemony and control within the sport can impact negatively upon Indigenous Fijians as well as (as we have seen) working-class and less well-connected Fiji Indians. We are unlikely to read in any FFA publications the statement by Vuilabasa that “[t]he [Indigenous] people feel that the game does not belong to them,” because it would detract from the upbeat corporate narrative promulgated by the FFA, including how the FFA desires to present soccer as a multicultural sport. These comments are not a denial of the facts that, very often, soccer *has* bridged boundaries between ethnic groups and social classes within Fiji or that it has left an undeniable positive footprint. Things are more complex than a simple inverted version of the FFA narrative would suggest.

### Issues relating to the FFA Veterans' Dinner 2014

In a promising initiative, the FFA hosted a Veterans' Dinner in Nadi on Saturday, October 4^th^, 2014, during the time of the Masters and regular IDCs. I attended this function as a guest of Dyer. The ex-players received a takeaway dish of noodles and a stubby (small bottle) of Fiji Bitter. Later, I asked Dyer why the Ba players were sitting by themselves on one side of the hall and not interacting with the Nadi players, except for Ba's Lote Delai who was talking with Dyer. The reason was that the ex-players were from different eras and didn't know each other, with the ex-Ba players being younger than the ex-Nadi players. While the ex-Nadi players in attendance had playing careers in the 1970s and early 1980s, the ex-Ba players present were district team representatives in the late 1980s and 1990s. None of our Ba interviewees—Bola, Sami, Tabaiwalu, and Vuilabasa—were in attendance that night. When interviewing these players in 2015, I asked them one-by-one whether they had received a formal invitation to the dinner or not. It seems that only Bola had received an invitation:

Meli: The Ba officials did not inform us about the Fiji FA Veterans' dinner last year. Fiji FA looks at the economic side of things but not the players' welfare.
Henry: Did you know about the Veterans' Dinner organized by Fiji Football?
Semi: No, I did not know about it, I was not given a ticket. After it happened, someone mentioned that something was happening in Nadi around the veterans' tournament.
Henry: He found out after the tournament had happened. Joe Basudra and Lote [Delai] (they had been at the function after match) were there, so why were you not there? They were younger players, after your time.
Semi: I was not informed until I learnt from somebody later on.
Henry: Inia Bola mentioned that he got an invitation. Do you think it is fair that he goes without your knowledge?
Semi: For me, it's OK. I have nothing to lose. Inia and me we are the same. But the question is why nobody ever told me.

Inia “Golden Boot” Bola, a star striker in the early 1980s for Ba and Fiji, was invited to the dinner, but he lashed out at the FFA for failing to consider his personal circumstances, living in poverty in the countryside at the back of Ba town. The journey to the dinner would have required a taxi fare and two bus journeys to travel the sixty kilometers to Nadi, with the prospects of finding an affordable ride back at that late hour fairly remote. “Do they think I am a taxi driver?” he asked rhetorically. In July 2019, the FFA inducted Bola as a “Legend,” along with three other ex-players, and it is possible that the FFA read our interview with Bola on the internet and decided to take corrective action. (Dyer certainly believes this to be the case.)

Author: Do you mean the 2014 Veterans' Dinner in Nadi?
Inia: Yes. They just gave me a ticket to the dinner but no transport. We live far away and many of us don't work; we could not make it.
Author: The officials don't understand the way of life and the problems faced by the Fijians if they just give them tickets but don't assist them to go to the dinner.
Henry: The officials today do not think about how far inland people are or whether they are bedridden, crippled or not working. They don't find out how the guys are. They just pass over the tickets.
Inia: My ticket was passed to me by Semi Tabaiwalu. Rajesh Patel [president of FFA] did not even arrange transport for us. It would have been great for the Ba boys from my era to have been at that function. I hope that this will not happen again. The former Fiji reps should be given free tickets at least for the IDCs.

The Fiji Indian ex-Ba player, Julie Sami, also declared unequivocally that he was not invited to the dinner in interview with the author:

Henry: Were you invited to the Fiji FA Veterans' Dinner last year 2014?
Julie: No.
Henry: Did you know they were inviting Inia Bola?
Julie: Nobody told me.
Henry: They didn't invite Semi [Tabaiwalu], we asked him about it. They invited the younger-generation players and Inia Bola, it's a mystery. Maybe they wanted to show the public that they invited Inia Bola because of the car accident.

It is a strange set of events that, out of these four ex-Ba players, only Bola knew about the dinner in advance. The younger ex-Ba players seemed to know about the dinner and a number of them were present in Nadi. Furthermore, not providing free transport and meals (other than the one provided at the dinner) shows that, at least at that time, the FFA was not giving sufficient consideration to the poverty and ill-health of many of the ex-players who were unable to travel the 60 km there and the 60 km back under their own steam. This information about who was invited to the dinner qualifies also as subjugated knowledges because it is unlikely to make its way into any official and sanitized accounts of Fiji soccer history produced or sponsored by the regulatory bodies or media.

### The story of the Nadi legends football club

The NLFC is a social club and self-help NGO set up by a group of ex-Nadi players and officials in 2004. Mahend Singh was instrumental in the formation of the club. Its purpose was and is to visit ex-players who are going through sickness, bereavement, or other difficulties and try to lend a helping hand and word of encouragement. The organization was designed to fill the gap left by the fact that FFA and the Nadi Soccer Association do little officially in terms of supporting ex-players. (Readers will recall the lawyer's tea-bag analogy from earlier in the article.) The ex-Airport Soccer Club and Nadi Soccer Association president, Bobby Tikaram, is active in this organization. The visits to ex-players are promoted by Tikaram and others in the “International Nadi Community” Facebook group. This group appears to be made up mostly of Fiji Indian émigrés based in Western countries.

Dyer as follows, in personal interview, explained to the author how the Nadi players of his era, after retirement, had no further contact with the Association for a number of years. The Association made no major efforts to contact or include the ex-players so they drifted away to pursue other pursuits. Then the idea of the NLFC emerged to fill the vacuum as it was decided that they had to begin their own efforts and work actively to support each other independently of the Association. They should no longer look to it for help. The name was chosen because no other sporting or social club in Nadi went by that name. In Dyer's words:

Most of us soccer players who had played together for Nadi in the same era just completely dropped out of soccer commitment. It was like a guillotine or an axe on our shoulder or on our back. I can't remember any of us being active with the Nadi Soccer Association after that. We just completely dropped out and lost contact with each other. We did not go to watch Nadi games unless there was a tournament here in Nadi and there was an invitation. We had the feeling that we had done our part and that was it. It might be mentioned by the Nadi Soccer Association that there were coaching training clinics but there were no personal follow-ups. We all just forgot about soccer until we formed the Nadi Legends Football Club in 2004.

One significant event was the visit to the home of former full-back Marika Vuniyawayawa 2 weeks before he passed away in the first half of 2014:

Marika was a veteran full-back for Nadi. He played many years before my playing career. We visited him 2 weeks before he passed away at his home. He had some kind of sickness in the throat and he could not speak well. However, at our appearance, he sat up just to meet us. He could hardly talk but he was talking with the fire from inside. This shows you how a veteran feels when he sees another colleague or mate coming to visit him in his time of need. As old friends, we told him to go and have his rest as we could see that he was struggling to talk to us. He said that he would not go to rest until our group had left his house, so we had to get away quickly! We had to leave without him knowing that we had gone so that he could have his rest. We had been doing this in the spirit of looking after each other during good times and bad times.

Dyer indicated to me that he has been trying to persuade other club members to set up a team in the Nadi club competition, following on from the footsteps of the earlier village-based Sweats Soccer Club (SSC), which fielded junior and senior teams for 5 or 6 years in the 1990s. This has not yet happened, and there may be a fear that to do so would mean coming out from under the radar and incurring the ire of controlling bodies. SSC lost its major sponsor and had to pull out of the Nadi club competition and Dyer remains upset today about the lack of moral and financial support forthcoming from the Association at the time. In Dyer's words:

In the sense of sporting fair play, Nadi Soccer Association also did not do much to help. If they were really happy about the Fijian guys forming a club they would have come to see us and worked out ways to help. They waited for us to drown. They did not offer us a hand to escape the deep water. They possibly thought that we were too good for the other clubs and so they began to work for the other clubs. The other village-based club Tanoa had nose-dived too. There are many soccer clubs in Nadi which died for financial reasons including Airport, Union, Young Ones, and a few others. Nadi Soccer Association did not have the insight to give them amnesty periods of 2 years of no fee payments to keep these clubs alive nor did they give the clubs advice about what they should do. It's a pity that there have been no village-based teams in the competition from that time up until today. We are trying our best to resurrect a Fijian-based soccer club.

If we look at all our interview quotes and accompanying interpretation, especially that of the ex-players who formed SSC and then NLFC, what conclusions can we draw? If we return to Foucault [(17), p. 83], he asks, with what were the subjugated knowledges, both the erudite and the disqualified, really concerned? He answers his own question (emphasis original): “with a *historical knowledge of struggles*.” The SSC was a village-based club, and the last village-based club in the Nadi club competition (although Tanoa met its demise around the same time). The SSC had three Indigenous ex-Nadi district team players as coaches and the now deceased village headman as president—one cannot get more “village” than that. They were given little moral or financial support once their major sponsor left, claims Dyer, and he also claims that the Nadi Soccer Association felt that the SSC was too good for the other clubs.

The NLFC is also ex-player and ex-official-led, including some Fiji Indian administrators from the same 1980s era, but it is less threatening to the current Association as it does not field junior or senior teams and simply remains a self-help support body. Lessons of “discipline and punish” have been well-learned and Dyer appears to be a lone voice suggesting the fielding of a team. The NLFC name is thus more controversial and bold than the reality. This genealogy, or multiplicity of genealogies, maintains Foucault, is absolutely not an attempt to revisit the fact- vs.-theory distinction or present a certain type of positivism. In fact, the fact-interpretation distinction is kept hazy, as subjugated knowledges contain both as this present article shows. Foucault [(17), p. 83] defines genealogy as “the union of erudite knowledge and local memories which allows us to establish a historical knowledge of struggles and to make use of this knowledge tactically today.” This would appear to well describe the memories and interpretations of the memories presented here. Another case was the ex-player organized Jone Nakosia Veterans' Tournament, arranged in 2012–2013, to honor the family of the deceased Ba veteran Jone Nakosia. It involved four or five district teams, and, in Dyer's words, “Fiji Football felt that they had lost out on a marketing opportunity.” In 2014, an approved Masters IDC was launched by the FFA featuring teams from Fiji and overseas.

The genealogy should direct its struggle at “the effects of the power of a discourse” (p. 84), and we work toward that here in conjunction with the words of wisdom and vision of our interviewees. As a result of the present article, more marginalized voices have been heard.

### Foucault and the body

The productive power of bodies [(24), p. 166] is seen in the way in which Indigenous players' bodies are regulated, controlled, and normalized by the Fiji Indian coaches and administrators, but only during their playing careers. After that, they are immediately dropped, as Iqbal Khan's tea-bag analogy powerfully illustrates. They are not wanted as senior coaches or administrators, thus reinforcing the hegemonic discourse of mind = Indian/body = Indigenous plus working-class Fiji Indian. The Indigenous ex-players are relegated back to their home villages where they are placed under the village gaze (and may do the gazing themselves) and they are removed from the corporate/town world where they are no longer allowed to belong. The situation can be seen in old photos where Indigenous bodies are presented performing amazing feats, but can only be controlled properly for an instant as they stand still for the ubiquitous, disciplinary team photograph. The restlessness of their bodies appears evident as they instinctively resist this ordering. The NLFC shows grassroots, productive, positive power [(24), p. 166] for action in a way which bypasses and escapes the regulatory intent of the official regulatory bodies, at least for now. Their power is too grassroots, too underground, too difficult to contain. Who else will visit ex-players when they are sick? Bobby Tikaram's role as an ex-official in the NLFC is significant here—because he has always been highly respected, he is allowed to enter that contemporary world of the ex-players without compromising his Fiji Indian identity. And while sympathy for Inia Bola may have led to him being the only ex-Ba player of his era to be invited to the Veterans' Dinner, the poverty and invalid status of Bola and some of his Ba contemporaries saw FFA want to block them from consciousness as bodies now presenting in unacceptable “deviant” form.

## Conclusion

We have used Foucault's (17) writings to introduce and explore the idea of “subjugated knowledges,” which was the focus of Foucault's academic explorations into the worlds of the asylum and the prison. It is knowledge which is buried within categories and systematized presentations, and it is the knowledge that is spoken by marginalized voices. Here, we present voices of ex-players who discuss their own experiences as victims of marginalization, after the end of their playing careers, and we also present their criticisms of the attitudes and behaviors of the regulatory bodies. Indigenous Fijian ex-players complain about a lack of coaching and administrative careers in the sport, after their playing careers are over, and even our Fiji Indian interviewee criticizes the “racial feeling” in the sport. This ex-player is excluded from networks of social power due to his social class position rather than his race/ethnicity.

We think that Foucault's distinction between the two types of subjugated knowledges should not be seen as too rigid and some knowledge could be both at once. Primarily, we suppose, it is the second type we are talking about here as the interviewed working-class and underclass ex-players are marginalized, disrespected voices. But their views also escape the commercial, upbeat narrative of official FFA publications, and hence meet the first type as well.

The other side of the coin, not hitherto covered in much detail, is the unstinting efforts of the mostly Fiji Indian administrators, at FFA, district, and club levels, to keep the sport moving and continuing to function, offering four major district-level tournaments a year, as well as a second-division, in a country mired by military coups, poverty, distance (some districts are based in places other than the main island Viti Levu), emigration to the West, and uncertain and sometimes tense and overdetermined race relations. Mention should be made of the moral and financial support offered by the Fiji Indian Diaspora in Western countries. They often return to Fiji for holidays and to visit family and to attend local soccer tournaments. They spend money and take updated knowledge of the soccer back overseas. They deify the soccer heroes of the past, and the players interviewed in this article would all be well-known and revered names in the Diaspora. James and Nadan (7) coined the terms “offshore memory” and “offshore library” to denote the knowledge cataloging of the sport by the Diaspora overseas.

## Data availability statement

The raw data supporting the conclusions of this article will be made available by the authors, without undue reservation.

## Ethics statement

The studies involving human participants were reviewed and approved by University of Fiji. The patients/participants provided their written informed consent to participate in this study. Written informed consent was obtained from the individual(s) for the publication of any potentially identifiable images or data included in this article.

## Author contributions

All authors listed have made a substantial, direct, and intellectual contribution to the work and approved it for publication.

## Conflict of interest

The authors declare that the research was conducted in the absence of any commercial or financial relationships that could be construed as a potential conflict of interest.

## Publisher's note

All claims expressed in this article are solely those of the authors and do not necessarily represent those of their affiliated organizations, or those of the publisher, the editors and the reviewers. Any product that may be evaluated in this article, or claim that may be made by its manufacturer, is not guaranteed or endorsed by the publisher.
